# Comparison Between Primary Anastomosis Without Diverting Stoma and Hartmann’s Procedure for Colorectal Perforation: A Retrospective Observational Study

**DOI:** 10.7759/cureus.58402

**Published:** 2024-04-16

**Authors:** Ryosuke Omoto

**Affiliations:** 1 Surgery, Toyonaka Municipal Hospital, Toyonaka, JPN

**Keywords:** retrospective observational study, diverting stoma, primary anastomosis, hartmann’s procedure, colorectal perforation

## Abstract

Background

Hartmann’s procedure (HP) is performed for colorectal perforation to avoid the risk of anastomotic leakage. Few reports have compared the safety between primary anastomosis without diverting stoma (PAWODS) and HP for colorectal perforation, and whether PAWODS or HP should be performed has remained controversial. We aimed to investigate the feasibility and safety of performing PAWODS in comparison to HP for colorectal perforation.

Methods

The data of 97 consecutive patients with colorectal perforation who underwent surgery from April 2010 to December 2020 were collected retrospectively. PAWODS and HP were performed in 51 and 46 patients, respectively. Univariate and multivariate analyses were performed to compare the clinical characteristics and postoperative outcomes of patients treated with PAWODS with those treated with HP.

Results

In the multivariate analysis, low serum albumin (hazard ratio (HR)=3.49; 95%CI=1.247-9.757; P=0.017) and left-sided colon and rectum perforation (HR=16.8; 95%CI=1.792-157.599; P=0.014) were significantly associated with the decision to perform HP. There was a significant difference in the mortality of the two groups (PAWODS vs. HP: 0% vs. 8.7%; P=0.047). The severe morbidity rate (Clavien-Dindo III-V) was significantly higher in the HP group (PAWODS vs. HP: 10% vs. 30%; P=0.020). In the PAWODS group, anastomotic leakage occurred in five of 51 patients (9.8%), four (8.7%) of whom required re-operation.

Conclusions

In appropriately selected patients, PAWODS could be safely performed with an acceptable rate of anastomotic leakage. The serum albumin level and site of perforation may be simple and useful factors for guiding decision-making on the surgical procedure.

## Introduction

Colorectal perforation is a serious disease that requires emergency surgery. Hartmann's procedure (HP) has been considered to be the most common procedure for avoiding the risk of anastomotic leakage [1,2]. However, recently, several randomized controlled trials (RCTs) have shown that primary anastomosis is superior to HP with regard to the stoma reversal rate and 12-month stoma-free survival with no significant differences in postoperative morbidity or mortality, even in cases involving perforated diverticulitis with purulent or fecal peritonitis classified as Hinchey III-IV [3-6]. In these RCTs, however, diverting stoma was constructed in most cases in the primary anastomosis group, and the factors that can be used to identify patients who potentially do not require diverting stoma are not fully understood [3-5].

For cases in which a diverting stoma is constructed, the operation time is extended and stoma complications (e.g., stoma stenosis or parastomal hernia) may occur [7]. Therefore, primary anastomosis without diverting stoma (PAWODS) is a more favorable surgical procedure if it is safely performed, but there have been no RCTs comparing the safety of PAWODS and HP. There are only three retrospective studies comparing the safety of PAWODS and HP for colorectal perforation [7-9]. In clinical settings, the decision as to whether to perform PAWODS or HP is decided during the operation by surgeons; however, the appropriate indications for PAWODS are not fully understood. In this study, to clarify the indications for cases in which PAWODS can be performed safely, we retrospectively investigated factors used to decide surgical procedures, and the short-term outcomes of PAWODS.

This article was previously posted to a preprint server (Research Square: https://www.researchsquare.com/article/rs-3344093/v1) on September 15, 2023.

## Materials and methods

Study design and patients

**Figure 1:**
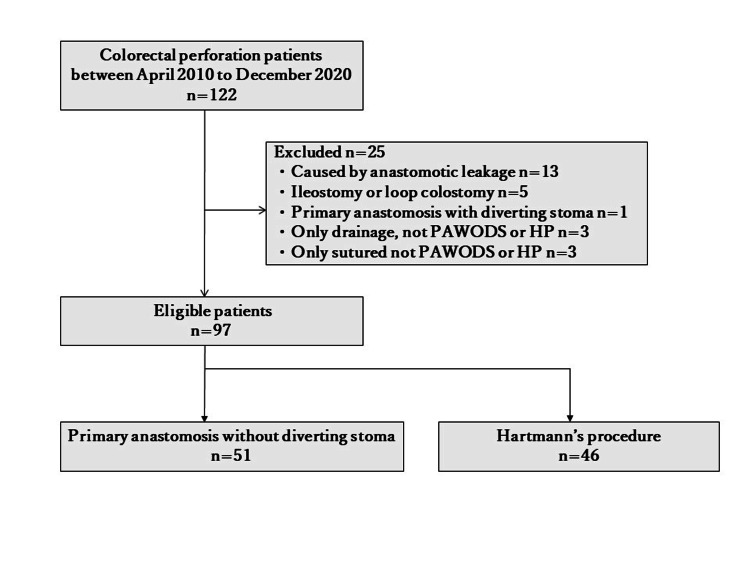
Flow diagram of patient disposition PAWODS: primary anastomosis without diverting stoma; HP: Hartmann’s procedure

This present single-center, retrospective study included 122 consecutive patients who underwent surgery for colorectal perforation in Toyonaka Municipal Hospital, Toyonaka, Osaka, Japan, between April 2010 and December 2020. After excluding 25 patients (anastomotic leakage, n=13; construction of ileostomy or loop colostomy, n=5; primary anastomosis with diverting stoma, n=1; and only drainage or primary suture, n=6), 97 patients who received PAWODS (n=51) or HP (n=46) were analyzed (Figure 1). The study was approved by the Toyonaka Municipal Hospital (approval number: 2021-07-02).

In all cases, the preoperative diagnosis of colorectal perforation was based on CT findings. Perforation was definitively diagnosed by pathological findings or by macroscopically apparent perforation in all cases. The surgical procedures were not randomized but were determined for each case by experienced gastroenterological surgeons based on preoperative vital signs, laboratory data, and intraoperative intra-abdominal findings. In all cases, patients were admitted to the intensive care unit (ICU) after surgery, and blood tests were performed before surgery and just after entering the ICU. 

Data collection

Medical records were retrospectively reviewed, and patient information, including sex, age, body mass index (BMI), and American Society of Anesthesiologists' physical status classification (ASA-PS), was collected. We also recorded perioperative outcomes, including the operation time, site of perforation, cause of perforation, severity of intra-abdominal pathology, and postoperative complications. We defined the cecum to the transverse colon as the right-sided, and the descending colon to the rectum as the left-sided. We calculated acute physiological and chronic health evaluation (APACHE) II and sequential organ failure assessment (SOFA) scores to evaluate the severity of sepsis [10,11]. The APACHE II score was calculated preoperatively, and the SOFA score was calculated intraoperatively with reference to anesthesia records. The severity of intra-abdominal pathology was intraoperatively graded using the Hinchey classification (I, II were localized, III was purulent, and IV was fecal peritonitis) [6]. Postoperative complications were graded using the Clavien-Dindo Classification (CDC), with CDC grades of III-V defined as severe [12].

Statistical analysis

All statistical analyses were performed using the JMP Pro 15 software program (SAS Institute, Cary, North Carolina, United States). In the univariate analysis, categorical variables were compared using Fisher’s exact test. Continuous data are reported as the median and interquartile range (IQR). Comparisons of continuous variables were examined using the Wilcoxon rank test. P values of <0.05 were considered statistically significant, and factors with a P value of <0.05 in the univariate analysis were entered into the multivariate analysis.

## Results

Patient characteristics

Table 1 shows all the patient characteristics. The median (IQR) age was 72 (61-82) years, 32% of patients had an ASA-PS score of III or worse, and the median serum albumin level was 3.1 (2.5-3.7) mg/dL. The median (IQR) APACHE II and SOFA scores were 7 (5-11) and 4 (1-5). There were 17 (18%) patients with right-sided perforation and 80 (82%) with left-sided perforation. Thirty-two (33%) had a Hinchey Classification of III and 26 (27%) had a Hinchey Classification of IV. Laparoscopic surgery was performed in 28 patients (29%).

**Table 1:** Patient characteristics (N=97) IQR, interquartile range; BMI, body mass index; ASA-PS, American Society of Anesthesiologists physical status classification system; APACHE, acute physiological and chronic health evaluation; SOFA, sequential organ failure assessment

Table 1. Patient characteristics	Values
Sex, n (%)	
Male	49 (51)
Female	48 (49)
Age (year), median (IQR)	72 (61-82)
BMI, median (IQR)	21 (19-23)
ASA-PS, n (%)	
Ⅰ	1 (1)
Ⅱ	65 (67)
Ⅲ	25 (26)
Ⅳ	4 (4)
Ⅴ	2 (2)
Serum albumin level (mg/dL), median (IQR)	3.1 (2.5-3.7)
APACHE Ⅱ, median (IQR)	7 (5-11)
SOFA, median (IQR)	4 (1-5)
Site of perforation, n (%)	
Right-sided colon	17 (18)
Left-sided colon and rectum	80 (82)
Hinchey-classification, n (%)	
Ⅰ,Ⅱ	39 (40)
Ⅲ	32 (33)
Ⅳ	26 (27)
Surgical approach, n (%)	
Open	69 (71)
Laparoscopic	28 (29)
Cause of perforation, n (%)	
Diverticulitis	57 (60)
Malignant tumor	16 (16)
Constipation	3 (3)
Ischemia	4 (4)
Foreign body	3 (3)
Iatrogenic	7 (7)
Other reasons	7 (7)

Predictive factors for PAWODS

The factors associated with the performance of HP in the univariate analysis of the PAWODS and HP groups are shown in Table 2. Age (PAWODS vs. HP: 67 (55-78) years vs. 79 (69-85); P<0.001), APACHE II score (6 (3-8) vs. 9.5 (7-13); P<0.001) and SOFA score (2.5 (1-4) vs. 4 (3-6); P<0.001) were significantly higher in the HP group than in the PAWODS group. The percentage of male patients (PAWODS vs. HP: 61% (n=31) vs. 39% (n=18); P=0.044) and serum albumin level (PAWODS vs. HP: 3.4 (2.8-3.8) mg/dL vs. 2.7 (2.2-3.2); P<0.001) were significantly lower in the HP group than in the PAWODS group. The rate of left-sided perforation (PAWODS vs. HP: 69% (n=35) vs. 98% (n=45); P<0.001) was significantly higher in the HP group than in the PAWODS group. In the multivariate analysis, a serum albumin level of ≤3.1 mg/dL (hazard ratio (HR)=3.49 (95%CI=1.247-9.757) P=0.017) and left-sided perforation (HR=16.8 (95%CI=1.792-157.599) P=0.014) were identified as independent predictive factors for the performance of HP (Table 3).

**Table 2:** Univariate analysis of predictive factors for HP HP, Hartmann's procedure; PAWODS, primary anastomosis without diverting stoma; IQR, interquartile range;  BMI, body mass index; IQR, interquartile range; APACHE, acute physiological and chronic health evaluation; SOFA, sequential organ failure assessment

Variables	PAWODS (N=51)	HP (N=46)	P-value
Age (year), median (IQR)	67 (55-78)	79 (69-85)	<0.001
Sex, n (%)			
Male	31 (61)	18 (39)	0.044
Female	20 (39)	28 (61)	
BMI, median (IQR)	21 (19-23)	21 (18-23)	0.583
Serum albumin level (mg/dL), median (IQR)	3.4 (2.8-3.8)	2.7 (2.2-3.2)	<0.001
APACHE-Ⅱ, median (IQR)	6 (3-8)	9.5 (7-13)	<0.001
SOFA, median (IQR)	2.5 (1-4)	4 (3-6)	<0.001
Site of perforation, n (%)			
Right-sided colon	16 (31)	1 (2.2)	<0.001
Left-sided colon and rectum	35 (69)	45 (98)	
Operation time (minutes), median (IQR)	183 (149-248)	203 (177-258)	0.233
Hinchey-classification, n (%)			
Ⅰ,Ⅱ	25 (49)	14 (30)	0.161
Ⅲ	15 (29)	17 (37)	
Ⅳ	11 (22)	15 (33)	
Surgical approach, n (%)			
Open	32 (63)	37 (80)	0.073
Laparoscopic	19 (37)	9 (20)	

**Table 3:** Multivariate analysis of predictive factors for HP HP, Hartmann's procedure; HR, hazard ratio; CI, confidence interval; APACHE, acute physiological and chronic health evaluation; SOFA, sequential organ failure assessment

Variables	HR	95%CI	P-value
Age ≥ 70 years	2.41	0.816-7.129	0.111
Sex (Male)	0.69	0.507-4.155	0.487
Serum albumin level (mg/dL)≤3.1	3.49	1.247-9.757	0.017
APACHE-Ⅱ≥9	1.99	0.669-5.910	0.216
SOFA≥4	1.82	0.598-5.507	0.292
Site of perforation (Left-sided colon and rectum)	16.80	1.792-157.599	0.014

Morbidity and mortality

The rates of postoperative morbidity and mortality are shown in Table 4. The overall morbidity rate of the PAWODS and HP groups did not differ to a statistically significant extent (PAWODS vs. HP: 53% vs. 54%; P=1.000); however, the severe morbidity rate of the PAWODS group was significantly lower than that of the HP group (PAWODS vs. HP: 10% vs. 30%; P=0.020). The organ space surgical site infection (SSI) rate in the PAWODS group was significantly lower than that in the HP group in the overall population (3.9% vs. 22%; P=0.012) and in the patients with a severe CDC grade (0% vs. 15%; P=0.004). Five of 51 patients (9.8%) in the PAWODS group developed anastomotic leakage, four of whom (8.7%) required re-operation. No death occurred in the PAWODS group, while four deaths (8.7%) occurred in the HP group (P=0.047); the causes of death were sepsis leading to multiple organ failure (n=3) and venous thromboembolism (n=1). The median length of the hospital stay was significantly shorter in the PAWODS group in the HP group (PAWODS vs. HP: 13 (10-22) days vs. 23 (17-39); P=0.020).

**Table 4:** Postoperative mortality and morbidity CDC, Clavien-Dindo classification; PAWODS, primary anastomosis without diverting stoma; HP, Hartmann's procedure; SSI, surgical site infection; VTE, venous thromboembolism

	All grades				CDC Grade Ⅲ to Ⅴ		
	PAWODS (n=51)	HP (n=46)	P-value		PAWODS (n=51)	HP (n=46)	P-value
Mortality n, (%)	0	4 (8.7)	0.047		-	-	-
Morbidity n, (%)							
Overall	27 (53)	25 (54)	1.000		5 (10)	14 (30)	0.020
SSI	14 (27)	17 (37)	0.385		0	11 (24)	<0.001
Incisional	13 (25)	13 (28)	0.821		0	4 (8.7)	0.047
Organ space	2 (3.9)	10 (22)	0.012		0	7 (15)	0.004
Anastomotic leakage	5 (9.8)	-	-		4 (8.7)	-	-
Anastomotic leakage (re-operation)	4 (8.7)	-	-		4 (8.7)	-	-
Ileus	6 (12)	3 (6.5)	0.492		1 (3.9)	1 (2.2)	1.000
Colon obstruction	2 (3.9)	0	0.496		0	0	1.000
Pneumonia	3 (5.9)	4 (8.7)	0.705		0	2 (4.3)	0.222
VTE	1 (2.0)	4 (8.7)	0.187		0	1 (2.2)	0.474
Others	6 (12)	10 (22)	0.273		1 (3.9)	3 (6.5)	0.343

Risk factors for anastomotic leakage

The risk factors for anastomotic leakage in the PAWODS group are shown in Table 5. Anastomotic leakage did not occur in patients with right-sided perforation, but we found no significant differences in the sites of perforation between the PAWODS and HP groups (P=0.155). The rate of anastomotic leakage in patients treated with the double stapling technique (DST) tended to be higher than that of patients who received functional end-to-end anastomosis (FEEA); however, the difference was not statistically significant (P=0.354).

**Table 5:** Predictive factors for anastomotic leakage in PAWODS PAWODS, primary anastomosis without diverting stoma; AL, anastomotic leakage; IQR, interquartile range; APACHE, acute physiological and chronic health evaluation; SOFA, sequential organ failure assessment; DST,double stapling technique; FEEA, functional end-to-end anastomosis

Variables	AL (-) (n=46)	AL (＋) (n=5)	P-value
Age (year), median (IQR)	67 (54-78)	63 (55-78)	0.966
Sex, n (%)			
Male	29 (63)	2 (40)	
Female	17 (37)	3 (60)	0.369
Serum albumin level (mg/dL), median (IQR)	3.4 (2.8-3.8)	3.4 (2.9-3.7)	0.881
APACHE-Ⅱ, median (IQR)	6 (3-8)	8 (3-10)	0.766
SOFA, median (IQR)	3 (1-5)	1 (0-5)	0.546
Site of perforation, n (%)			
Right-sided colon	17 (37)	0	0.155
Left-sided colon	29 (63)	5 (100)	
Hinchey-classification, n (%)			
Ⅰ,Ⅱ	24 (52)	1 (20)	0.169
Ⅲ	12 (26)	3 (60)	
Ⅳ	10 (22)	1 (20)	
Surgical approach, n (%)			
Open	29 (63)	3 (60)	1.000
Laparoscopic	17 (37)	2 (40)	
Operation time (minutes), median (IQR)	179 (149-249)	220 (143-258)	0.773
Anastomotic method, n (%)			
DST	23 (50)	4 (80)	0.354
FEEA	23 (50)	1 (20)	

## Discussion

In the present study, firstly, we evaluated the factors that impacted the surgeon’s decision on the surgical procedure. In the univariate analysis, age, sex, serum albumin level, APACHE II score, SOFA score, and the site of perforation were identified as predictive factors for deciding the surgical procedure. In the multivariate analysis, the serum albumin level and the site of perforation remained as independent predictive factors for deciding the surgical procedure. The serum albumin level reflects the preoperative nutritional status and general condition [13,14]. A low serum albumin level is a risk factor for anastomotic leakage and is a risk factor for mortality during sepsis due to vascular hyperpermeability [15-19]. In the current study, the serum albumin level of the HP group was significantly lower than that of the PAWODS group, which means that surgeons had appropriately selected HP for patients with poor conditions. This result was similar to previous studies; the HP group was significantly older, more hemodynamically unstable, and more immunosuppressive than the PAWODS group [8,9]. APACHE II and SOFA scores are other indicators of the general condition that are useful for predicting the severity and prognosis of sepsis [10,11]. Although we speculated that the APACHE II and SOFA scores could be associated with decision-making in relation to the surgical procedures, these factors were not associated with the surgical procedures. Furthermore, regarding the simplicity of calculation, the serum albumin level is much simpler in comparison to the APACHE II and SOFA scores which require complicated calculations. This study shows that serum albumin level can be a simple and useful index as a factor in determining the surgical procedure. In addition, there may be a possibility of avoiding unnecessary stoma or anastomotic leakage resulting from forced PAWODS.

Considering the postoperative results, the HP group had significantly higher mortality and morbidity rates than the PAWODS group. Zingg et al. reported that in the HP group, the mortality and morbidity rates were significantly higher and that the baseline characteristics were significantly worse in comparison to the PAWODS group [8]. On the other hand, Tsuchiya et al. revealed that the 30-day mortality and re-operation rates were significantly higher in the PAWODS group than in the HP group; however, in that study, the baseline characteristics were adjusted using propensity score matching [7]. Thus, although it may be risky to perform PAWODS for all patients with colorectal perforation, it may be safely performed with equivalent mortality and morbidity rates to HP in appropriately selected patients. In the present study, the favorable postoperative outcomes of PAWODS also supported that the intraoperative decision for surgical procedure was appropriate.

The incidence rate of anastomotic leakage was 9.8% (5/51) and 7.8% (4/51) of patients required re-operation. These rates of anastomotic leakage were similar to the reported incidence rates of 4-14% in patients who underwent primary anastomosis with or without diverting stoma for perforated diverticulitis [3,4,20-22]. However, in these studies, 50-100% of patients who underwent primary anastomosis were performed also diverting stoma [3,4,21]. The rate of anastomotic leakage in patients who received PAWODS for perforated diverticulitis has only been reported in one study and the rate of anastomotic leakage of any grade was 28.3% [8]. Therefore, we thought that the anastomotic leakage rate of 9.8% was acceptable. This acceptable anastomotic leakage rate may also imply that we could appropriately assign patients to PAWODS and HP groups intraoperatively.

We investigated the risk factors for anastomotic leakage in patients treated with PAWODS. No anastomotic leakage occurred in patients with right-sided colon perforation and only patient treated with FEEA developed anastomotic leakage. Although the right-side location and FEEA tended to be associated with less anastomotic leakage in comparison to left-side location and DST, we could not find significant differences between these groups. Although we thought that the location and procedure of anastomosis were strong candidate factors, these factors were not significant factors in the present study. These results could be due to the weak statistical power of the present study as a result of its relatively small population. Notably, 16 patients (94%) with right-sided colon perforation received PAWODS and none of these patients developed anastomotic leakage. A previous study of right-sided colon perforation also reported that none of the 34 patients with right hemicolectomy developed anastomotic leakage [23]. Although serum albumin level is known to be associated with anastomotic leakage [15], we found no significant association. As described above, the serum albumin levels in HP patients were significantly lower than those in PAWODS patients. Therefore, after the exclusion of HP patients with relatively lower serum albumin levels, the serum albumin level may not have remained an independent risk factor for anastomotic leakage among patients with non-low serum albumin levels. In appropriately selected patients, PAWODS may be a safe surgical procedure that is associated with an acceptable rate of anastomotic leakage. In particular, we thought that PAWODS could be safely performed for patients with right-sided colon perforation.

The present study was associated with some limitations. First, this was a single-center, retrospective observational study with a relatively small population; thus, selection biases may have existed and the results might have been affected by the retrospective design. A larger sample size and prospective study are necessary to determine the surgical procedure (HP or PAWODS). Second, we included right-sided colon perforation. There are only two retrospective studies of right-sided colon perforation [23,24]. However, the results obtained after the exclusion of right-sided cases did not differ from the results of analyses that included the right-sided cases (data not shown). In our study, the rate of anastomotic leakage in patients with left-sided perforation was 14%, and this may also be acceptable in comparison to previous studies [8]. Third, 39 patients (40%) had localized peritonitis classified as Hinchey I-II, and this rate was relatively high in comparison to previous studies [3-5,25].

## Conclusions

Our study indicated that in the appropriately selected patients, we could safely perform PAWODS for colorectal perforation. The preoperative serum albumin level (≤3.1mg/dL) and location of perforation is a simple and useful factor for guiding decision-making on the surgical procedure. However our study is small sample size and retrospective, A larger sample size and prospective study is necessary to determine the surgical procedure (HP or PAWODS).
